# Copeptin as a marker of atherosclerosis and arteriosclerosis

**DOI:** 10.1016/j.atherosclerosis.2021.10.012

**Published:** 2021-11-01

**Authors:** Fredrika Schill, Margaretha Persson, Gunnar Engström, Olle Melander, Sofia Enhörning

**Affiliations:** a Department of Cardiology, Skåne University Hospital, Carl-Bertil Laurells gata 9, 214 28, Malmö, Sweden; b Department of Clinical Sciences, Lund University, Jan Waldenströms gata 35, 214 28, Malmö, Sweden; c Department of Internal Medicine, Skåne University Hospital, Jan Waldenströms gata 11 A, 214 28, Malmö, Sweden

**Keywords:** Vasopressin, Copeptin, Atherosclerosis, Arteriosclerosis, Coronary calcium score, Pulse wave velocity

## Abstract

**Background and aims:**

The precursor peptide of vasopressin, copeptin, has previously been linked to increased risk of developing diabetes mellitus, coronary artery disease and cardiovascular mortality. Whether elevated copeptin is associated with markers of atherosclerosis and arteriosclerosis in the general population is not known.

**Methods:**

In this population-based, cross-sectional study, coronary artery calcium score (CACS), carotid-femoral pulse wave velocity (c-f PWV) and fasting plasma copeptin were measured in 5303 individuals in the Swedish cardiopulmonary bioimage study (SCAPIS). Multivariable logistic regression models were used to analyze the associations between copeptin and high CACS (>100) and high c-f PWV (>10 m/s), respectively.

**Results:**

The number of individuals with high CACS and c-f PWV increased across increasing tertile of copeptin (11.7%, 13.3% and 16.3% for CACS and 6.9%, 8.5% and 10.6% for c-f PWV). The top tertile of copeptin was, compared with reference tertile 1, significantly associated with both high CACS and high c-f PWV after adjustment for age, sex, hypertension, diabetes mellitus, HDL, triglycerides, BMI, smoking status, creatinine and high sensitive CRP with an odds ratio (OR) of 1.260 (95% confidence interval (CI): 1.022–1.555) for CACS and OR 1.389 (95% CI: 1.069–1.807) for PWV.

**Conclusions:**

Copeptin is associated with both coronary atherosclerosis and increased arterial stiffness in the general population. Our data indicates that copeptin may be a useful marker in the assessment of cardiovascular risk.

## 1 Introduction

The pituitary hormone vasopressin (VP) is known for its regulation of blood osmolality together with its vasopressor and hemostatic effects. With broadened knowledge about its vastly distributed three receptors, vasopressin has lately been acknowledged for its importance in several other physiological systems [1]. The vasopressin system has, through measurement of its stable marker copeptin, been shown to be upregulated in several cardiometabolic conditions. Elevated copeptin concentration in plasma is associated with the metabolic syndrome, hypertension and microalbuminuria [2,3]. Furthermore, elevated copeptin can predict development of diabetes mellitus, diabetic heart disease, chronic kidney disease, coronary artery disease and cardiovascular mortality [4–7], as well as predict both prognosis and development of heart failure [8–12].

Cardiovascular disease (CVD) is the leading cause of death world-wide [13], and is usually a consequence of atherosclerosis-related organ damage. Atherosclerosis is a component of arteriosclerosis and is defined as the luminal narrowing of arteries by deposition of plaques [14]. Arteriosclerosis is a remodeling process predominantly affecting the elastic arteries, which is commonly called arterial stiffness. There are several methods of evaluation of the burden of atherosclerosis and arteriosclerosis; two of them being measurement of coronary calcium score (CACS) and pulse wave velocity (PWV).

CACS is an estimation of coronary artery calcification. CACS is identified as areas of hyper-attenuation on computed tomography (CT) [15] and is highly correlated with the total coronary atherosclerosis load [16]. Elevated CACS has been shown to predict CVD and death in several studies [15,17]. Furthermore, CACS improves cardiovascular risk classification in addition to traditional risk factors [18,19] and can be used to guide therapy according to current guidelines [20–23]. Several studies have proposed CACS >100 as an appropriate cut-off for predicting CVD risk [18,24].

PWV is a marker of arterial stiffness. Loss of vessel distensibility results in an increased PWV, which is calculated by dividing the distance between two sites of an arterial segment by the arterial pressure wave transit time. When assessing carotid-femoral PWV (c-f PWV), the right common carotid and right femoral arteries are used [14]. C-f PWV has been established as the gold standard method for measuring arterial stiffness [25,26]. A c-f PWV value exceeding 10 m/s is considered elevated [27]. C-f PWV is shown to be an independent predictor of coronary heart disease, stroke and CVD [14], and is, like CACS, suggested as a risk reclassification method among individuals with a moderate cardiovascular risk [23].

Even though a link between copeptin and several cardiovascular conditions has been established, a possible association between copeptin and objective measures of subclinical CVD has not been investigated. This study aims to determine whether copeptin is associated with markers of atherosclerosis and arteriosclerosis measured by CACS and PWV.

## 2 Materials and methods

### 2.1 Population description

SCAPIS is a collaborative project between six Swedish universities investigating the cardiopulmonary health of a random selection of individuals aged 50–64 years, residing in the six respective cities (Gothenburg, Linköping, Malmö/Lund, Stockholm, Umeå and Uppsala). Between 2013 and 2018, 30,154 men and women were recruited. The participants completed an extensive questionnaire and underwent a health examination including anthropometry, blood sampling and CT with measurement of CACS. The overall participation rate was 50%. PWV measurement was included in the baseline examination for participants examined in Malmö. A total of 6251 individuals were examined at this site. Out of these, complete data including PWV and CACS, copeptin and cardiovascular risk factors were available in 5303 individuals. The research was conducted in accordance with the Declaration of Helsinki. The multicenter study was ethically approved by the Umeå University Ethical Review Board whereas the present study was approved by the Lund University Ethical Review Board. All participants provided written informed consent [28].

### 2.2 Examinations

C-f PWV was measured with Sphygmocor Xcel (Atcor Medical, Australia) in supine position after 5 min of rest with cuffs on the upper left arm and on the right thigh approximately 10–20 cm below the groin. The carotid-femoral distances were measured from the femoral pulse to the upper edge of the thigh cuff and from the carotid pulse to the upper edge of the thigh cuff. A carotid tonometer was then used simultaneously with the leg cuff to capture blood pressure waveforms at the carotid and femoral sites. Two measurements of c-f PWV were then obtained. If the difference between the measurements differed more than 0.5 m/s, a third measurement was performed. The mean value of the measurements was used in further analysis. The calcium content in each coronary artery, visualized by CT (Somatom Definition Flash, Siemens Medical Solution, Forchheim, Germany) was measured and summed to produce a total CACS [28] according to the scoring system previously described by Agatston et al. [29]. Weight was measured with participants in light clothing, using calibrated scales. Blood pressure was measured in the brachial artery of both arms after 5 min of supine rest and calculated as the average of two stable measurements (difference <10 mmHg) of the arm with the highest value. Prevalent hypertension was defined as having a brachial systolic blood pressure ≥140 mmHg, diastolic blood pressure ≥90 or use of antihypertensive medication. Prevalent diabetes mellitus was defined as fasting plasma glucose ≥7.0 mmol/L, HbA1C ≥ 48 mmol/mol or use of antidiabetic medication.

### 2.3 Laboratory measurements

A venous blood sample was collected from participants after an overnight fast, and laboratory analyses (cholesterol, high-density lipoprotein (HDL), triglycerides (TG), calculated low-density lipoprotein (LDL), high sensitive c-reactive protein (hsCRP) and creatinine) were performed at the Department of Clinical Chemistry, Skåne University Hospital, Malmö, Sweden.

Biobanked (- 80 °C) fasting plasma samples were used for analysis of copeptin. Copeptin was successfully analyzed in 5779 individuals using a KRYPTOR Compact Plus device and a commercially available chemiluminescence sandwich immunoassay copeptin ProAVP kit with coated tubes from samples stored at –80 °C (BRAHMS Copeptin proAVP KRYPTOR; ThermoFisher Scientific).

### 2.4 Statistical analysis

Baseline characteristics were expressed as absolute counts and proportions for categorical variables and as means ± standard deviations (SD) for continuous variables. The distribution of copeptin was not normally distributed and was therefore transformed using the natural logarithm. Tertiles of copeptin, with sex-specific tertile limits, were used throughout. PWV and CACS were examined as categorical variables. HsCRP was categorized into quartiles due to a considerable number of participants with values below the lower limit of quantification (LLQ, i. e., 0.6 mg/L). Chi-square test was used to assess between group differences of categorical variables and Student’s t-test was used to investigate between group differences of continuous variables. Multivariable logistic regression models were used to assess the associations between copeptin and high CACS (>100) and PWV (>10 m/s), respectively. All models were adjusted for age and sex and in the next step additionally adjusted for prevalent hypertension, prevalent diabetes mellitus, BMI, smoking status, HDL, TG, creatinine and hsCRP.

The level of significance was set at *p* < 0.05. All analyses were performed using the SPSS statistical software (version 25.0; SPSS, Chicago, Illinois, USA).

## 3 Results

The present study sample consisted of 5303 individuals in which complete data on fasting plasma copeptin, PWV and CACS was available. The mean age was 57.5 years and 46.7% were men. Baseline characteristics of the participants, presented in groups of high and low CACS and PWV, respectively, are shown in Table 1. Individuals with high CACS were to a higher extent men and smokers, had a higher age and BMI, had more often a diabetes diagnosis and high blood pressure, lower HDL and higher TG, creatinine and CRP concentrations than individuals with lower CACS, whereas the LDL levels did not differ significantly between individuals with high *versus* low CACS (Table 1). Individuals with high PWV were to a higher extent men, had a higher age, had more often a diabetes diagnosis and high blood pressure, lower HDL and higher TG, creatinine and CRP concentrations than individuals with lower PWV, whereas smoking status, BMI or LDL levels did not differ significantly between individuals with high *versus* low PWV (Table 1).

The median copeptin concentration was higher within the groups of high CACS and high PWV as compared to the groups of lower CACS and PWV in both men and women, although it did not reach significance for PWV among women (*p* = 0.062) (Table 1).

A total of 13.7% had high CACS and 8.6% had high PWV. Across increasing sex-specific tertile of copeptin, the proportion of high CACS was 11.7%, 13.3% and 16.3% (*p* < 0.001), and the proportion of high PWV was 6.9%, 8.5% and 10.6% (*p* = 0.001). Increasing tertile of copeptin was significantly associated with both high CACS and high PWV, after adjustment for age and gender (Table 2). The associations remained significant after adjustment of additional cardiovascular risk factors (hypertension, diabetes mellitus, BMI, smoking status, HDL, TG, creatinine and hsCRP) (Table 2). The associations between copeptin and cardiovascular risk factors are shown in Supplemental Table 1.

Among the 948 individuals with incomplete data, the mean age was 57.5 (4.3) years and 47% were men. Data on CACS were available in 703 individuals out of which 14.1% had high CACS, whereas data on PWV were available in 623 individuals out of which 9.7% had high PWV.

## 4 Discussion

In this population-based cross-sectional study with high participation rate, the key finding is that elevated copeptin concentration in plasma is significantly associated with CACS and PWV above the cut-off values considered as pathological, also after adjustment of several cardiovascular risk factors. The median copeptin concentration was significantly higher in the groups of high CACS and PWV, except for PWV among women. The latter could be a consequence of loss of power, since the number of women in the group of high PWV was relatively small (n = 122).

The mechanisms behind the link between vasopressin/copeptin and CVD are not known. Previous studies have shown that elevated copeptin predicts development of CVD, cardiovascular mortality, incidence and prognosis in heart failure as well as several cardiovascular risk factors [2,5–8,10,11]. Arteriosclerosis is mainly a disease of the larger elastic arteries such as the aorta and is, more than atherosclerosis, linked to hypertension and age [30]. We have previously found a cross-sectional association between copeptin and hypertension [2], but in the current study the significant association between copeptin and PWV remained significant after adjustment for hypertension.

Elevated copeptin is shown to predispose for new onset diabetes mellitus [5,31], and a causal relationship between the vasopressin system and diabetes mellitus has been proposed by a human Mendelian randomization study [32]. Further, through the pituitary vasopressin 1b receptors, vasopressin is known to mediate adrenocorticotrophic hormone (ACTH) release which in turn elevates glucocorticoid levels. The ACTH release has been reported to be resistant to glucocorticoid feedback in contrast to the CRH-induced ACTH release [33]. This would suggest that excessive vasopressin release may induce a mild Cushing-like phenotype with overweight, obesity, insulin resistance and increased cardiovascular risk, as indicated by data from several epidemiological studies [2,3,5–7,31].

Another possible role of vasopressin in CVD development is promoting inflammation. Vasopressin is a pro-inflammatory peptide and stimulates the release of several cytokines and other inflammatory peptides, hereby inducing an inflammatory cell response. Interestingly, immunoneutralization of serum vasopressin in rats has been shown to diminish inflammation [1]. Many studies linking copeptin to different cardiovascular risk factors or conditions have adjusted for c-reactive protein (CRP) in an intent to eliminate the possibility that inflammation is driving the observed association. However, since inflammation is highly complex, measurement and adjustment for CRP are not sufficient to rule out inflammation as the possible link between vasopressin and CVD.

### 4.1 Clinical implications

Both CACS (>100) and PWV (>10 m/s) can be used as risk assessment tools for CVD and are suggested to be of value in the risk reclassification of moderate CV risk patients [23]. However, even if the radiation dose of a CACS CT can be relatively low (<1 millisievert, mSv), the dose varies up to as high as 8 mSv [34]. This, together with its demands on health care resources, restricts the availability of the exam [21]. PWV measurement is seen neither as a practical nor an effective risk evaluation method and is therefore not recommended in routine use [35]. Biomarkers as risk assessment tools are continuously growing as an entity due to lack of negative aspects such as radiation and restricted availability/efficiency. Copeptin has previously been suggested as a biomarker for prediction of outcome and prognosis of several cardiovascular conditions, such as myocardial infarction, heart failure and stroke [36–38]. Our current results point at copeptin as a potential risk marker of both atherosclerosis and arteriosclerosis. Possibly, it could also be used as an easy-to-measure blood biomarker for individuals with intermediate cardiovascular risk based on traditional risk factors, in order to select candidates for further risk assessment with CACS or CT-angiography.

### 4.2 Limitations

This cross-sectional cohort study has several limitations. First, due to its cross-sectional design, it is not possible to draw any conclusions regarding causality. Secondly, complete data was lacking in a substantial number of individuals (N = 948), which could possibly obscure the results. However, baseline characteristics of this group (age, sex, proportion of high CACS and high PWV) were comparable to baseline characteristics of the study sample. Thirdly, the cut-offs used (PWV >10 and CACS >100) is not equal to established CVD, and many individuals with high CACS or PWV never develop CVD. As well, coronary plaques that are not calcified does not elevate CACS. Whether copeptin can discriminate CV risk better than CACS and/or PWV is an interesting area of further studies.

### 4.3 Conclusion

Copeptin is associated with markers of atherosclerosis and arteriosclerosis. We propose a possible role of copeptin as a non-invasive, low risk and cost-effective method of evaluating CV risk.

## Supplementary Material

Supplementary material

## Figures and Tables

**Table 1:** Baseline characteristics stratified by Coronary Artery Calcium Score (N = 5303) and Pulse Wave Velocity (N = 5303).

	CACS ≤100 n = 4574	CACS >100 n = 729	*p*-value CACS ≤100 *vs*. CACS >100	PWV ≤10 n = 4845	PWV >10 n = 458	*p*-Value PWV ≤10 *vs*. PWV >10
Age, years^a^	57.2 (4.3)	59.5 (4.0)	<.0.01	57.2 (4.2)	59.9 (3.9)	<0.001
Men, n^b^	1951 (43.7)	528 (72.4)	<0.01	2142 (44.2)	336 (73.4)	<0.001
LDL, mmol/L^a^	3.6 (0.9)	3.7 (1.1)	0.093	3.6 (0.9)	3.6 (1.0)	0.63
HDL, mmol/L^a^	1.7 (0.5)	1.5 (0.5)	<0.001	1.7 (0.5)	1.5 (0.5)	<0.001
TG, mmol/L^a^	1.2 (0.7)	1.5 (1.0)	<0.001	1.3 (0.8)	1.5 (0.8)	<0.001
SBP, mmHg^a^	122.2 (16.3)	129.7 (16.8)	<0.001	121.4 (15.4)	142.5 (16.2)	<0.001
DBP, mmHg^a^	75.0 (9.7)	77.7 (9.9)	<0.001	74.5 (9.3)	84.2 (9.7)	<0.001
BMI, kg/m^2^ ^a^	26.9 (4.3)	28.1 (4.3)	<0.001	27.0 (4.3)	27.4 (4.3)	0.07
Smoking, n^b^	723 (15.8)	168 (23.1)	<0.001	79 (17.2)	812 (16.8)	0.79
Prevalent diabetes mellitus, n^b^ (%)	278 (6.1)	110 (15.1)	<0.001	303 (6.3)	85 (18.6)	<0.001
Prevalent hypertension, n^b^ (%)	1257 (27.5)	360 (49.4)	<0.001	1306 (27.0)	311 (67.9)	<0.001
Copeptin, pmol/L^c^	4.9 (3.5–7.6)	6.6 (4.3–9.8)	<0.001	5.0 (3.6–7.7)	6.6 (4.3–10.7)	<0.001
Males	6.8 (4.7–10.1)	7.5 (5.1–10.7)	0.002^d^	6.7 (4.7–10.1)	7.5 (5.4–11.8)	0.001^d^
Females	4.0 (3.1–5.7)	4.3 (3.3–6.5)	0.024^d^	4.0 (3.1–5.8)	4.3 (3.3–6.3)	0.062^d^
Creatinine, μmol/L^a^	76.9 (14.8)	80.2 (15.4)	<0.001	77.1 (14.9)	80.4 (15.3)	<0.001
hsCRP, mg/L^c^	1.1 (0.6–2.3)	1.2 (0.7–2.8)	0.002^d^	1.1 (0.6–2.3)	1.4 (0.7–2.8)	<0.001^d^

CACS, Coronary Artery Calcium Score; PWV, pulse wave velocity; LDL, low density lipoproteins; HDL, high density lipoproteins; TG, Triglycerides; Hba1C, glycated hemoglobin SBP, brachial systolic blood pressure, DBP, brachial diastolic blood pressure; BMI, body mass index; hsCRP, high sensitive c-reactive protein.

a Values are mean ± (Sd).

b n (%).

c median (IQR).

d Mann-Whitney *U* Test.

**Table 2:** High coronary calcium score (>100) and high pulse wave velocity (>10 m/s) in tertiles of copeptin.

	Copeptin tertile 1	Copeptin tertile 2	Copeptin tertile 3	*p* trend
Copeptin, pmol/L^a^				
Males	4.14 (1.43–5.42)	7.03 (5.44–8.91)	12.54 (8.93–456.7)	
Females	2.83 (0.65–3.44)	4.08 (3.45–5.07)	6.96 (5.08–407.4)	
High coronary calcium score	N = 1800	N = 1783	N = 1720	
Adjustment^b^		1.159 (0.943–1.426)	1.449 (1.185–1.772)	<0.001
Adjustment^c^		1.081 (0.874–1.335)	1.264 (1.024–1.559)	0.027
High pulse wave velocity	N = 1800	N = 1783	N = 1720	
Adjustment^b^		1.227 (0.951–1.583)	1.538 (1.203-1.967	0.001
Adjustment^c^		1.198 (0.918–1.563)	1.392 (1.070–1.809)	0.014

BMI, body mass index; TG, triglycerides; HDL, high density lipoproteins; hsCRP, high sensitive c-reactive protein.

a Expressed as median (minimum – maximum).

b Adjusted for age and gender.

c Adjusted for age, gender, BMI, TG, HDL, prevalent diabetes mellitus, hypertension, smoking, creatinine, hsCRP.
