# Supplementary material for: Copeptin as a marker of atherosclerosis and arteriosclerosis
Source: Atherosclerosis. Author manuscript; Available in PMC 2022 Feb 9. (PMC7612343; doi:10.1016/j.atherosclerosis.2021.10.012)
Supplement: Supplementary material [file EMS140804-supplement-Supplementary_material.doc]

| **Supplemental table 1.** Copeptin in relation to cardiovascular risk factors. | | | |
| --- | --- | --- | --- |
|  | **Beta-coefficient** | **Confidence interval** | ***p*-value** |
| Age, years | 0.001 | -0.004 -0.005 | 0.724 |
| Gender, male | 0.421 | 0.372 – 0.470 | <0.001 |
| HDL, mmol/L | 0.028 | -0.017 - 0.073 | 0.217 |
| TG, mmol/L | 0.031 | 0.002 – 0.060 | 0.038 |
| BMI, kg/m² | 0.015 | 0.009 – 0.020 | <0.001 |
| Smoking | 0.063 | 0.011 – 0.114 | 0.017 |
| Prevalent diabetes mellitus | 0.016 | -0.062 – 0.095 | 0.680 |
| Prevalent hypertension | 0.035 | -0.008 – 0.078 | 0.108 |
| Creatinine, µmol/L | 0.004 | 0.003 – 0.006 | <0.001 |
| hsCRP, increasing quartile | 0.018 | 0.002 – 0.034 | 0.024 |
| Values are given as beta-coefficient (95% CI) of increase of LN-transformed plasma copeptin concentration in relation to unit increase in the – or presence of the – independent variable. | | | |
